# Extended-Spectrum Beta-Lactamase-Producing *Escherichia coli* and Other Antimicrobial-Resistant Gram-Negative Pathogens Isolated from Bovine Mastitis: A One Health Perspective

**DOI:** 10.3390/antibiotics13050391

**Published:** 2024-04-25

**Authors:** Breno Luis Nery Garcia, Stéfani Thais Alves Dantas, Kristian da Silva Barbosa, Thatiane Mendes Mitsunaga, Alyssa Butters, Carlos Henrique Camargo, Diego Borin Nobrega

**Affiliations:** 1 Department of Animal Nutrition and Production, School of Veterinary Medicine and Animal Science, University of São Paulo, Pirassununga 13635-900, SP, Brazil; brenoluis.garcia@ucalgary.ca (B.L.N.G.); stefani.nutri@gmail.com (S.T.A.D.); barbosa.kristian@usp.br (K.d.S.B.); thatiane.mitsunaga@usp.br (T.M.M.); 2 Faculty of Veterinary Medicine, University of Calgary, Calgary, AB T2N 1N4, Canada; alyssa.butters@ucalgary.ca; 3 Bacteriology Division, Adolfo Lutz Institute, São Paulo 05403-000, SP, Brazil; carlos.camargo@ial.sp.gov.br

**Keywords:** dairy cattle, public health, AMR genes, interspecies transmission

## Abstract

Antimicrobial resistance (AMR) poses an imminent threat to global public health, driven in part by the widespread use of antimicrobials in both humans and animals. Within the dairy cattle industry, Gram-negative coliforms such as *Escherichia coli* and *Klebsiella pneumoniae* stand out as major causative agents of clinical mastitis. These same bacterial species are frequently associated with severe infections in humans, including bloodstream and urinary tract infections, and contribute significantly to the alarming surge in antimicrobial-resistant bacterial infections worldwide. Additionally, mastitis-causing coliforms often carry AMR genes akin to those found in hospital-acquired strains, notably the extended-spectrum beta-lactamase genes. This raises concerns regarding the potential transmission of resistant bacteria and AMR from mastitis cases in dairy cattle to humans. In this narrative review, we explore the distinctive characteristics of antimicrobial-resistant *E. coli* and *Klebsiella* spp. strains implicated in clinical mastitis and human infections. We focus on the molecular mechanisms underlying AMR in these bacterial populations and critically evaluate the potential for interspecies transmission. Despite some degree of similarity observed in sequence types and mobile genetic elements between strains found in humans and cows, the existing literature does not provide conclusive evidence to assert that coliforms responsible for mastitis in cows pose a direct threat to human health. Finally, we also scrutinize the existing literature, identifying gaps and limitations, and propose avenues for future research to address these pressing challenges comprehensively.

## 1. Introduction

Bovine mastitis is one of the most prevalent diseases of dairy cattle and the main reason for antimicrobial use (AMU) on dairy farms in North America [1,2]. Treatment and prevention of intramammary infections (IMIs) will account for up to 80% of total AMU on farms [3]. Broadly, the goal of antimicrobial therapy is 3-fold: (1) eliminate the causative pathogen responsible for clinical mastitis (CM), (2) treat existing IMIs at drying off, and (3) prevent new IMIs from occurring during the dry period. The effectiveness of this approach is contingent upon many factors, with the causative agent playing a pivotal role [4].

Improvements in mastitis control programs within modern dairy herds have led to a notable decrease in the prevalence of pathogens causing contagious mastitis. Regrettably, this was followed by increased incidence rates of opportunistic Gram-negative bacteria, which now account for nearly 40% of the CM cases in well-managed herds [5]. The benefits of antimicrobial therapy of mild and moderate cases of CM caused by Gram-negative pathogens, particularly coliforms, have not been well established [6]. Many antimicrobials used for CM treatment have limited activity against Gram-negative pathogens. Additionally, IMIs caused by *Eschericha coli* (*E. coli*), one of the most important and prevalent Gram-negative bacteria causing mastitis, responds poorly to antimicrobial therapy and have high rates of spontaneous cure [7]. Consequently, antimicrobial intervention is not always warranted.

Antimicrobial resistance (AMR) is an increasingly urgent global health issue intricately related to antimicrobial use (AMU) in both human and veterinary medicine [8]. Although associations between AMU in animal production and AMR in humans are not always evident, the alarming rate in which antimicrobials are consumed in food-producing animals leads to a sustained pressure for reducing AMU in livestock [9]. Livestock production accounts for nearly 70% of the medically important antimicrobials sold in the United States [8], including critically important antimicrobials (CIA). Importantly, the World Health Organization (WHO) defines CIAs as limited drugs important to treat life-threatening human infections for which bacteria may have transmitted from non-human sources or acquired resistance genes from non-human sources [10]. Consequently, resistance against CIAs in bacteria from livestock is worrisome and needs to be critically evaluated.

*E. coli* is also an important cause of infections in humans. Notably, extended-spectrum beta-lactamase-producing *Escherichia coli* (ESBL-Ec) is resistant to a broad range of antimicrobials that are commonly used in clinical settings. Infections caused by ESBL-Ec can be challenging to treat, leading to prolonged illness, increased healthcare costs, and a higher risk of treatment failure [11]. ESBL-Ec strains are known for their ability to transfer resistance genes horizontally to other bacteria. This horizontal gene transfer can occur both within the same species and between different species. This capacity for genetic exchange contributes to the spread of AMR in various environments, including healthcare settings, communities, and livestock. Therefore, the emergence and spread of ESBL-Ec in livestock could potentially represent a risk to humans, recognizing the interconnectedness of human, animal, and environmental health.

Our goal in this review is to have a critical discussion about the role of antimicrobial-resistant Gram-negative pathogens causing CM towards human health, with a focus on ESBL-Ec and *Klebsiella* spp. This review will discuss characteristics of antimicrobial-resistant strains causing CM and human infections, elements associated with their presence, molecular mechanisms of AMR in bacteria from mastitis, interspecies transmission as well as recommended interventions strategies. We will also delve into the limitations of the current literature and suggest future directions for research.

## 2. Epidemiology

### 2.1. Antimicrobial-Resistant, Gram-Negative Pathogens from Bovine Mastitis and Humans

Gram-negative coliforms are common agents of bovine mastitis [12]. The high prevalence of coliforms in CM is related to their environmental characteristics. Coliforms are commonly present in the environment of dairy farms [13]. Among coliforms, *E. coli* is the most prevalent pathogen causing CM on farms, followed by *Klebsiella* spp. Both pathogens have demonstrated a concerning trend of escalating incidence rates in recent years [5].

Specific strains such as *E. coli* sequence type (ST) 131 and *Klebsiella pneumoniae* ST258 have emerged as important pathogens causing infections in humans and were responsible for a rapid increase in AMR rates of clinically important pathogens worldwide [14] (Table 1). *E. coli*, with its remarkable genetic diversity, exhibits versatility as both a commensal and opportunistic pathogen. Pathogenic *E. coli* can be divided according to the site of infection and origin of isolates. Those strains that cause intestinal infections in humans are called intestinal pathogenic *E. coli* (InPEC) and can be subdivided into different pathotypes (e.g., enterohaemorrhagic [EHEC], enteroinvasive [EIEC], enteroaggregative [EAEC], shiga toxin-producing [STEC]) [15]. Pathogenic strains that have the potential to cause extraintestinal infections are called extraintestinal pathogenic *E. coli* (ExPEC). ExPEC strains include uropathogenic *E. coli* (UPEC), meningitis-associated *E. coli* (MNEC), avian pathogenic *E. coli* (APEC), mammary pathogenic *E. coli* (MPEC), and others [16]. This classification is primarily based on genetic or phenotypic determinants of virulence coupled with the strain’s ability to cause infections [17].

Human *E. coli* infections will have a wide range of clinical manifestations. *E. coli* can cause enteric infections with severity associated with the occurrence of different pathotypes and their virulence genes [18]. Among extraintestinal infections, *E. coli* ranks as the most frequent Gram-negative pathogen isolated from bloodstream infections (BSI) in hospitalized patients, with increasing detection rates of ESBL [19]. *E. coli* is also the most prevalent pathogen causing urinary tract infections, with complicated infections associated with the more virulent uropathogenic *E. coli* (UPEC) pathotype [20]. Regarding AMR, several mechanisms that can confer resistance to virtually any drug can be present in *E. coli* [21].

*E. coli* pathotypes correlate moderately with the phylogenetic background (or phylogroup) and with the ST as determined by multilocus sequence typing. For instance, ExPECs ST131, ST73, and ST95 belong to phylogroup B2 [15], which harbors many strains that are virulent to humans. Conversely, most (65–80%) MPEC strains are associated with phylogroups A or B1 [22,23,24,25,26,27,28], demonstrating limited genetic overlap with virulent human strains.

Specific strains of *E. coli* are responsible for the majority of human infections worldwide [29]. *E. coli* ST131 is one of the most globally disseminated antimicrobial-resistant clone [30]. ST131 is composed of several clades: clade A is mostly linked to fimH41, clade B to fimH22, and clade C to fimH30. Clade C is most related with AMR and is further divided into three subclades, C0/1/2. The C2/fimH30-Rx group within ST131 is the major cluster responsible for the spread of CTX-M-15 in humans worldwide, whereas the C1/H30R is responsible for CTX-M-27 distribution. To date, presence of these strains in the dairy cattle population is very rare. ST131 is not common among environmental and veterinary isolates and appears to be a human-adapted pathogen, as previously suggested [11].

The ability of specific clades to colonize and infect humans was likely influenced by modifications in fimH alleles. Prior to the 1990s, clade B was prevalent. However, during the 2000s, clade C took the lead, accounting for up to 80% of all ST131 infections worldwide [11]. The establishment and spread of this clade in a community context may have been significantly impacted by the apparent effectiveness of *E. coli* ST131 transmission amongst members of the same family [14]. Considering ESBL enzyme types, the CTX-M-lactamase is most prevalent globally among nosocomial and community isolates, being present worldwide in various species and especially common in *E. coli*, while the NDM, OXA-48, and KPC are the most frequent carbapenemases [14].

*Klebsiella pneumoniae*, another clinically important Gram-negative bacteria that causes diseases in humans [31], is also a frequent agent of bovine mastitis. Mastitis caused by *Klebsiella* spp. are increasingly prevalent and respond poorly to antimicrobial therapy [32]. The most common clinical manifestation of infections caused by *K. pneumoniae* in humans is lobar pneumonia, often acquired in the community. This infection is characterized by a severe and acute-onset manifestation, with a high mortality rate, contrasting with more subtle cases of bronchopneumonia or bronchitis, usually hospital-acquired [18]. In addition, other hospital-associated infections include urinary tract infections and BSIs. For BSIs, global studies have ranked *K. pneumoniae* as the 3rd most frequent pathogen over the last 20 years [19]. Furthermore, AMR is prevalent in *K. pneumoniae* of human origin. The presence of ESBLs is as high as 30% among BSI isolates recovered from several regions in the world [19]. Since the 1990s, carbapenemases have emerged and disseminated among Enterobacterales, including *K. pneumoniae* [33]. Currently, carbapenem-resistant Enterobacterales (CRE) have been listed as critical priority pathogens by the World Health Organization.

In humans, specific clonal groups (CG) can be locally or globally disseminated and often demonstrate host specificity with regards to virulence and resistance determinants [31]. For instance, the global CG258 is recognized as one of the most resistant clones, exhibiting resistance to aminoglycosides, carbapenems, quinolones, and, more recently, to polymyxins [34]. ST258 is a dominant strain that has been circulating in more than 20 countries and is responsible for the fast and extensive spread of KPC-producing *K. pneumoniae* globally [35]. Although *bla*_KPC_ has been detected in more than 100 STs, strains from the clonal complex 258 (CC258) are predominant among KPC-producing *K. pneumoniae* in humans. In general, *K. pneumoniae* lineages from humans and non-human niches share limited genetic overlap [36], and no specific lineage has become dominant among ones causing mastitis in dairy cows [36]. Nevertheless, KPC-2-positive ST258 strains were recently detected among dairy cows with clinical mastitis in Mexico at an alarming rate [37]. *K. pneumoniae* from mastitis appears to have a low prevalence of ESBLs. A comprehensive genomic study involving 180 isolates from 11 different states in the US revealed that only four isolates (2%) were ESBL producers and associated with the presence of the *bla*_CTX-M-1_ gene [38].

As for *E. coli*, CTX-M-15-positive strains were detected in cattle samples from the UK, Brazil, France, and Japan [39,40]. Additionally, CTX-M and TEM ESBL types were detected in milk samples from Italy [41,42]. The prevalence of ESBLs in *E. coli* from mastitis is likely influenced by geographical location. In Brazil, *bla*_CTX-M-15_ was identified in 2 isolates out of 161, belonging to ST10 and ST993 [27]. In Europe, 12 isolates out of 878, classified as different STs, harbored *bla*_CTX-M-1_, *bla*_CTX-M-2_, *bla*_CTX-M-14_, and *bla*_CTX-M-15_ genes [24]. Likewise, in Switzerland, ESBLs were uncommon in *E. coli* from bulk tank milk or mastitis [43]. In China, however, higher ESBL frequencies were reported. Nearly 23% of MPEC isolates were ESBL-positive with a diverse range of associated genes, including *bla*_CTX-M-15_, *bla*_CTX-M-14_, *bla*_CTX-M-55_, *bla*_CTX-M-64_, *bla*_CTX-M-65_, and *bla*_CTX-M-3_ [44]. Another study from China also identified a 66% prevalence of ESBLs among 83 MPEC isolates, also attributable to CTX-M enzymes [45].

In general, there has been an uptick in literature reports detailing cases of mastitis caused by antimicrobial-resistant Gram-negative coliforms. This surge could stem from either a genuine rise in incidence or biases in reporting practices. In the following section, we delve into specific agricultural practices that have been linked to the prevalence of these resistant strains on farms.

### 2.2. Factors Associated with AMR in Gram-Negative Bacteria Causing Mastitis

Despite the continued use of antimicrobials in the dairy industry, there is not yet solid evidence supporting that AMR rates have increased among mastitis pathogens over time [46], which endorses the notion that associations between antimicrobial use and resistance in bacteria from mastitis are transient. Nevertheless, there are important risk factors for AMR in Gram-negative bacteria from mastitis or from the farm environment, most related to farm management and antimicrobial stewardship practices [47].

The use of third- or fourth-generation cephalosporins (ceftiofur, cefoperazone, and cefquinome) for the treatment of CM has been implicated as a contributing factor for the emergence of ESBLs among *E. coli* causing mastitis on dairy cows. ESBL isolates from milk were almost four times more common following CM treatment with third- or fourth-generation cephalosporins [47].

As for raw milk samples, environmental contamination and farm hygiene practices are typically associated with the presence of antimicrobial-resistant Gram-negative isolates. A study in China found similarities in the distribution of AMR rates in E. coli isolated between raw milk and environmental sources, suggesting that drug-resistant isolates from the environment were detected in contaminated milk samples [48]. Snow and collaborators demonstrated that inadequate hygiene of calf-feeding equipment and slurry management were risk factors for the presence of ESBL on farms. Furthermore, the high frequency of antimicrobial residues on replacement milk and the presence of ESBL-producing Enterobacteriaceae in calves’ feces were also associated with environmental contamination on farms [49].

## 3. Transmission

Given that resistance genes have the potential to spread throughout the environment and the food chain and that there is some (limited) overlap in STs detected in mastitis and humans, the presence of organisms harboring ESBLs, KPCs, and others in bovine milk raises significant concerns regarding the potential human health risks linked to these genetic elements. ESBL-producing *E. coli* isolates can be transferred between humans and animals directly through contact or indirectly through food or the environment [50,51]. It is assumed that dairy farm workers would be at increased risk of bacterial colonization and potential infection with ESBL-producing Enterobacterales from cows with mastitis through direct contact during milking; however, data supporting this are sparse [52,53]. The transmission of ESBL-producing organisms through dairy-derived food has also not been thoroughly investigated, complicated by variations in farm and food chain management practices and agricultural policies [52]. Molecular data indicate that animals are not a major source of the ESBL-Ec causing infections in humans, and human-to-human transmission should be the main focus of public health policy in order to avoid infections caused by antimicrobial-resistant bacteria in humans [54].

Overall milk quality also varies greatly worldwide, influenced by milk collection systems and cooling infrastructure [55]. Although effective pasteurization kills Enterobacterales in milk, ingestion of raw milk [52] as well as ineffective pasteurization of milk containing high bacterial loads [55] are areas of concern. Consumption of raw milk or raw cheese products varies greatly between countries, ranging from 3.2% (raw milk) and 1.6% (raw cheese) in the US [53] to 27% in Brazil [27]. The magnitude of the potential risk of AMR transmission to humans resulting from the consumption of dairy products is not well explored. While there is limited evidence that raw milk consumption may be associated with the spread of bacteria from cows to humans [32,56], most extraintestinal infections by Gram-negative bacteria arise from the gut [57]. Therefore, the risk of transmission associated with the consumption of raw milk will be dependent on the prevalence of these organisms in raw milk samples as well as on the ability of these strains to persist in the intestinal environment of humans following the consumption of contaminated milk. In that regard, the mere presence of specific human strains such as *K. pneumoniae* ST258 in milk samples is certainly of concern and should be further explored [37,55,56].

The dissemination of AMR genes among different isolates could occur via the exchange of mobile genetic elements (MGEs), such as insertion sequences (ISs), phages, transposons, and plasmids, which might carry supplementary AMR genes [24,58]. Therefore, inconsistency in the distribution of ESBL-producing Enterobacterales strains in bovine milk or causing bovine mastitis and those detected in human clinical strains does not necessarily preclude the transfer of resistance elements from animal-associated bacteria to human pathogens. The most concerning mechanisms of resistance, demonstrating a cross-border spread among animals, humans, and the environment, are indeed governed by mobile AMR genes. The orchestrated presence of AMR genes, virulence factors, mobile genetic elements, and other pathogenicity determinants is essential for the effective spread of these microorganisms in any environment [58,59]. We will next describe MGEs detected in isolates from bovine mastitis and contextualize these in relation to those identified in clinical human isolates.

### Mobile Genetic Elements of Gram-Negative, Antimicrobial-Resistant Isolates from Mastitis

Insertion sequences (ISs) are extremely simple MGEs, but the contribution of certain ISs to the widespread dissemination of resistance elements has been widely recognized [60]. Encoding at minimum a transposase gene that facilitates genetic mobility, ISs are delineated into groups based in part by the active site motifs in this transposase enzyme [60]. Several families of ISs have been linked to successful and efficient AMR dissemination. These include IS26, implicated in the transmission of many AMR genes among Gram-negative bacteria [60], and ISCR1 and ISEcp1, implicated in the pandemic expansion of ESBL-producing Enterobacerales in recent decades [61].

ISEcp1 has been frequently associated with *bla*_CTX-M_ genes and are commonly identified in an upstream location that facilitates two functions: providing a transposase and promoting the expression of downstream (ESBL) genes through the provision of a strong promoter [60,61]. Unlike other ESBL variants such as SHV and TEM, CTX did not evolve through mutation of existing genes but rather was acquired through horizontal gene transfer from an environmental organism, *Kluyvera* spp. [61,62]. The insertion of ISEcp1 upstream from the *bla*_CTX-M_ gene is postulated to have facilitated the movement of the ESBL gene onto a plasmid [63], which has since spread within Enterobacterales. It has also been noted in some ESBL isolates, however, that associated ISEcp1-*bla*_CTX-M_ are chromosomally located [64], indicating chromosomal reinsertion in some cases. ISEcp1 has also been associated with *bla*_CMY-2_- and *bla*_OXA-181_-like genes [60]. Studies on bacteria from mastitis have similarly identified this association between ESBLs and ISEcp1 [39,65,66,67,68]. Further, a common genetic arrangement of ISEcp1 *bla*_CTX-M_-orf477 (complete or truncated) was noted not only in bovine mastitis ESBL-producing isolates [67,68], but also in isolates from healthy humans and food-producing animals [45] as well as in ST131 from humans [69].

ISCR1 elements have also been linked to *bla*_CTX-M-2_ and *bla*_CTX-M-9_ genes [62]. ISCR1 has been reported as a frequent MGE among ESBL-producing isolates from mastitis in association with *bla*_TEM_ and *bla*_SHV_ in addition to *bla*_CTX-M_ [70], but that has been reported from a single study. Like ISEcp1, ISCR elements can also act to mobilize downstream genes, but may bear the ability to mobilize larger passenger elements due to their hypothesized rolling circle replication and ability to mediate the formation of complex Class I integrons [61,63].

Integrons represent another MGE that has contributed pivotally to the transmission of AMR due to their ability to capture, transmit, and express AMR genes [39,60,71]. Integrons consist minimally of three components: an integrase enzyme (encoded by an *intI* gene), an attI recombination site, and a promoter (Pc). The integrase gene can catalyze recombination between the attI site and a cognate site (attC) on a gene cassette, a small non-replicative element carrying typically only a single gene or two (often AMR genes) and lacking a promoter. Multiple gene cassettes of ARGs can be combined into gene arrays, with proximity to the Pc dictating the degree of expression of the gene. Integrons are denoted as class 1, 2, or 3, defined by the sequence of the *intI* gene [71].

Integrons are commonly reported in ESBL-producing *E. coli* and *Klebsiella* spp. isolates from normal or mastitic bovine milk and almost exclusively found to be class 1 [24,67,68,70,72]. The prevalence of class 1 integrons within ESBL isolates from these studies ranged from 40% [72] to 83.3% [70]. Common AMR found on cassettes within these integrons included *dfrA17*-*aadA5* [67,68,70,72], *dfrA1*-*aadA1* [24,68,72], *dfrA12*-*aadA2* [24,67], and others. Class 1 integrons are also highly prevalent among *E. coli* and *K. pneumoniae* isolates from hospitals, with 57% of isolates being positive for class 1 integrons [73]. *dfrA* and *aadA* genes are predominant [74] and are typically associated with specific STs of *K. pneumoniae* of human origin such as ST11, ST15, ST147, ST562, and ST716 [75]. The prevalence and notable diversity within integrons within ESBL-producing *E. coli* and *Klebsiella* spp. from mastitis emphasize the need to investigate the full genetic context of these MGEs when investigating epidemiological associations. Nevertheless, epidemiological investigations focusing on the attributable risk of human AMR due to the presence of class 1 integrons in mastitic isolates are currently non-existent.

Plasmids play a crucial role in facilitating the horizontal transfer of a large number of AMR genes, such as ESBL genes [39,76]. Plasmids are described as DNA molecules with a double-stranded structure, either circular or linear, endowed with the ability for independent replication and transferability among various bacterial species and clones. Most widely acknowledged plasmids are typically identified via their capability to confer phenotypes that undergo positive selection within the bacterial host. These phenotypes often involve the presence of genes associated with antimicrobial resistance or virulence. The acquisition of plasmids carrying such genes has the potential to significantly impact the prevalence of virulent or multidrug-resistant bacterial clones [77].

The horizontal transfer of plasmids among distinct bacterial isolates takes place via conjugation. Plasmids that are not inherently capable of self-transmission through conjugation can be mobilized at a high frequency when a helper plasmid is present [78]. Plasmid classification follows a formal scheme based on incompatibility (Inc) groups. Plasmids with identical replication controls are labeled as “incompatible”, while those with differing replication controls are regarded as compatible. Consequently, two plasmids belonging to the same Inc group cannot coexist within the same cell line [79].

Beta-lactam resistance is frequently encountered in isolates of *E. coli* and *K. pneumoniae* from cases of bovine mastitis [80]. Plasmid families show uneven distribution in clinically significant enterobacterial strains, with some families being more prevalent [78]. The replicon types IncF, IncI1, IncN, and IncHI1 emerge as the primary carriers of ESBL/AmpC genes and should be acknowledged as the prevailing ESBL/AmpC plasmids in food and food-producing animals. In any case, particular gene-plasmid combinations, exemplified by *bla*_CTX-M-1_/IncI1, *bla*_CTX-M-1_/IncN, or *bla*_CTX-M-15_/IncF, are known for their propensity to disseminate extensively in an epidemic manner [81]. According to Madec et al. [82], there is a significant prevalence of IncI1 plasmids in animals.

Cefotaxime-resistant *E. coli* isolates collected from cattle feces in 10 geographical areas (districts) in France during the period from 2007 to 2009 revealed a shared pool of *bla*_CTX-M-15_ plasmids between cattle and humans. These results highlight the potential role of plasmid-driven dissemination of ESBL genes between animals and humans [81].

Accordingly, several studies highlight CTX-M as the predominant beta-lactamase in *E. coli* isolated from bovine mastitis. In China, spanning from 2013 to 2017, the primary beta-lactamase gene among *E. coli* isolates from the milk of cows with mastitis was *bla*_TEM_, followed by *bla*_CTX-M_, *bla*_CMY_, and *bla*_SHV_. Additionally, in strains derived from mastitis milk, the most prevalent genes were *bla*_CTX-M-2_ in Japan, *bla*_CTX-M-14_ in France, and *bla*_CTX-M-15_ in the UK [59]. In their comprehensive analysis of ESBL/AmpC in *E. coli* isolates derived from samples collected in 35 studies spanning The Netherlands and in its different reservoirs, Dourado-Garcia et al. [51] noted that *bla*_CTX-M-1_ predominantly dominated in dairy cattle, veal calves, pigs, and the pig farming community, and was notably prevalent in retail beef and veal calf meat at the slaughterhouse.

Among bovine *E. coli* isolates from quarter milk samples of cows with or without mastitis in south-western Germany, three plasmids carrying the *bla*_CTX-M-15_ gene were identified, one belonging to IncI1, another to IncFIA + IncFIB, and one to IncF. Furthermore, IncHI2 plasmids were detected in a single isolate, coexisting with IncP [24,80]. The presence of the *bla*_CTX-M-15_ gene has been identified in numerous isolates of mastitis-related *E. coli* and *K. pneumoniae* worldwide [80].

Antimicrobial susceptibility data from *E. coli* and *K. pneumoniae* isolates in clinical cases of cattle mastitis from 2009 and 2011 identified in France were analyzed. Two *E. coli* isolates were identified as part of the ST23 clonal group, carrying the *bla*_CTX-M-1_ IncI1/ST87 and the *bla*_CTX-M-14_ F18:A-:B plasmids, respectively. Additionally, another two *E. coli* isolates were typed as ST58, one harboring the *bla*_CTX-M-1_ IncI1/ST3 plasmid and the other carrying the *bla*_CTX-M-14_ F2:A-:B- plasmid. The remaining *E. coli* isolates were ST10, which are rare among humans in France. *K. pneumoniae* isolates belonged to the ST45 group. It was also demonstrated that the *bla*_CTX-M-1_ genes were carried on IncI1 plasmids, while *bla*_CTX-M-14_ genes were located on IncF plasmids. Furthermore, F2:A-:B plasmids were found to carry only the *bla*_CTX-M-14_ gene and were identified in both *E. coli* and *K. pneumoniae* [39].

In their review on AMR of pathogens causing bovine mastitis globally, Naranjo-Lucena and Slowey [80] identified the presence of *bla*_OXA_ beta-lactamases in *E. coli* isolates from Lebanon, *bla*_CMY-2_ in Switzerland, Thailand, South Korea, and Lebanon, and *bla*_CMY-59_ in Canada. The transposon-associated *bla*_KPC_, capable of inserting itself into a plasmid, was detected in *K. pneumoniae* from Mexico. Isolates from India harbored the *bla*_NDM-1_ gene, commonly detected in human *E. coli* from the same geographical location [83]. *K. pneumoniae* from Pakistan carried both *bla*_NDM-1_ and *bla*_OXA-48_. The *bla*_NDM-5_ gene was identified in *E. coli* and *K. pneumoniae* from milk samples collected in Algeria and China, respectively.

MGEs harboring AMR genes can be seen in many shapes and forms, likely reflective of multiple recombination events [61,84]. Within a One Health framework, we believe that even the detection of the same MGEs within human and animal isolates is not sufficient to delineate potential transmission events, but provides some guidance for future investigations, particularly when contextualized with the magnitude of associated risks. The clustering of MGEs and AMR genes observed by Naranjo-Lucena and Slowey [80] hints at an evolution towards more human-predominant types in dairy cows that should be further explored. It is likely that the spectrum of antimicrobial use in animals and humans is also similar within countries. Furthermore, studies seldom, if ever, address the risk of interspecies transmission between humans and cows, leaving a significant gap in knowledge crucial for informing public policy.

Next, we discuss potential intervention strategies that could be implemented to facilitate future investigations focused on the transmission of antimicrobial-resistant Gram-negative isolates from mastitis origin between dairy cows and humans as well as reduce their prevalence in the dairy cattle population.

## 4. Intervention Strategies

### 4.1. Surveillance and Monitoring

ESBL-producing Enterobacteriaceae strains responsible for bovine mastitis have been identified across various regions globally [85]. Implementing robust surveillance systems for these organisms in mastitis-related milk samples could facilitate epidemiological investigations, particularly concerning their zoonotic potential. Moreover, genomic surveillance would greatly aid in conducting future source attribution studies.

Integrated surveillance programs that report on the presence of AMR in livestock animals, including dairy cattle, have already been implemented in a few parts of the world [86,87]. In Canada, the CaDNet program consists of coordinated data collection on farms in five regions across Canada. An annual collection of bulk tank milk and fecal samples is performed, together with a questionnaire to gather data on herd management techniques, AMU, and risk factors for AMR. Samples are collected by regional field workers and sent to a central laboratory to perform microbiological culture (targeting *E. coli*, *Campylobacter* spp., and *Salmonella* spp.) and minimum inhibitory concentration (MIC) analysis of isolates.

In Europe, the VetPath pan-European surveillance program is a remarkable example of a program that includes AMR surveillance of mastitis isolates. The mastitis component of VetPath consists of a coordinated milk sample collection of CM cases in eight participant countries: Belgium, Czech Republic, France, Germany, Italy, the Netherlands, Switzerland, and the United Kingdom [88,89]. Samples are collected and processed in each country under the responsibility of one national coordinator, following uniform guidelines for pathogen isolation. Each tested herd is limited to one sample each year in order to maximize the chance of examining strains that are not related epidemiologically. Isolates are then sent to a central laboratory where MIC analyses are performed in accordance with the Clinical and Laboratory Standard Institute (CLSI). According to the cefquinome MIC results, *E. coli* and *Klebsiella* spp. isolates are selected for identifying potentially ESBL/AmpC-producing isolates, based on genome sequencing [89].

Recent VetPath results pointed out to an overall low but increasing resistance profile among the *E. coli* isolates from CM [88,89]. Furthermore, prevalence of ESBL/AmpC producers was also observed [89]. Although these were not alarming rates in comparison to other livestock species [54], these trends were upsetting and need to be further investigated.

### 4.2. Antimicrobial Stewardship in Clinical Mastitis Treatment and Prevention

Antimicrobial stewardship is an important element in a multifaceted approach to combat AMR [86]. In general, modifying treatment protocols and on-farm practices is challenging [87]. Mastitis control practices aim to prevent the occurrence of new intramammary infections and eliminate existing cases of CM. For the latter, the following four possibilities typically apply: (a) spontaneous cure (e.g., the immune system is capable of eliminating the pathogen); (b) removal of cows with chronic mastitis; (c) antimicrobial treatment during lactation; or (d) dry cow therapy (DCT). Hence, antimicrobial therapy turns out to be one of the most important control strategies for CM on dairy farms [90].

While antimicrobial therapy is a crucial element of mastitis control programs, not all clinical cases will require the administration of antimicrobials [91], considering that about 50% of CM cases results in spontaneous cure or are caused by microorganisms not susceptible to antimicrobials [92,93]. Given that farm-level AMU is associated with AMR in Gram-negative mastitis-causing pathogens [2,47,94], this unnecessary use of antimicrobials will likely booster AMR rates on farms, particularly when specific antimicrobials (e.g., 3rd or 4th generation cephalosporins) are used in the treatment [47].

Clinical, bacteriological, and overall cure rates of CM can be used to measure the effectiveness of antimicrobial therapies [95]. These metrics are dependent on many factors, including the following: (a) factors associated with the cow; (b) antimicrobial treatment protocol (e.g., antimicrobial, route of administration, and treatment duration), and (c) the mastitis-causing agent. Ideally, decisions on whether antimicrobials should be administered or not to any specific case of CM should be based on all these elements. Some cow-related factors that affect the cure rates of CM include parity (e.g., older cows have a reduced likelihood of recovery), high SCC during lactation, and days in milk [4,95]. As for the mastitis etiology, some species, like *E. coli*, respond poorly to antimicrobial therapy and have high spontaneous cure rates [96], whereas others such as *Klebsiella* spp. might require antimicrobial therapy for observing increased bacteriological cure rates [97].

Considering that different approaches are recommended depending on the pathogen, the adoption of rapid, on-farm identification of organisms will be an effective way to implement selective treatment practices and reduce total AMU [98]. On-farm microbiological culture systems consist of using either selective, differential, or chromogenic culture media for performing on-farm identification of species or groups of the major mastitis-causing agents [99]. Results can be used to inform selective therapy of CM, guiding antimicrobial treatment decision [98]. Selective treatment of CM consists of treating with antimicrobials only those cases in which there is a benefit associated with the treatment, while observing other animals and intervening only if necessary [98,100]. Important elements to be considered when adopting selective CM treatment are, in addition to the microbiological identification of the causative agent, characteristics of the cow (e.g., SCC, days in milk, parity, and history of CM in the current lactation) and the CM severity score [6]. For mild and moderate cases of CM, selective treatment can reduce in about 50% of cases the use of antimicrobials [98,99], without decreasing bacteriological cure risks [100].

Furthermore, antimicrobials are frequently used on farms at drying off. In some countries, dry cow therapy (DCT) accounts for most AMU on dairy farms [101]. Blanket DCT consists of administrating antimicrobials to all cows at drying-off. Although this approach has been widely adopted [102], it necessarily implies administering antimicrobials to some healthy mammary quarters, resulting in the excessive use of antimicrobials. In this regard, mammary quarters or cows can be effectively selected for antimicrobial therapy at drying off, which can be an efficient strategy to reduce AMU on farms [103].

Another alternative for the control of mastitis caused by Gram-negative pathogens is vaccination. Currently available vaccines are generally effective in reducing the severity of CM cases caused by *E. coli* and *Klebsiella* spp. [104]. The vaccine for *E. coli* is based on the J5 strain [105], while for *Klebsiella* spp., two products are available: one based on siderophore receptor proteins [106] and another containing a recombinant protein (YidR) [107]. Although vaccines are beneficial for reducing the severity of CM cases, increasing milk production, and decreasing the risk of culling, no evidence is available to support their use for preventing new intramammary infections by Gram-negative pathogens [104].

### 4.3. Environmental Stewardship

Waste milk will inevitably contain antimicrobial residues and can potentially be one of the most important sources of antimicrobial-resistant bacteria causing CM on farms. Indeed, residues of medically important antimicrobials and multidrug-resistant bacteria including the multidrug-resistant *E. coli* were detected in waste milk [108,109].

Feeding preweaning calves with waste milk is a common practice on farms [110,111]. This practice has been associated with increased AMR levels in the microbiota of calves [112,113]. Waste milk treatment protocols (e.g., prolonged storage time, heat treatment, and electrochemical methods) have been suggested to mitigate the negative impacts of drug residues on waste milk; however, the efficacy of these methods is yet to be determined [112]. Heat treatment at 120° for 20 min promotes degradation of β-lactam antibiotics, with degradation rates depending on the antimicrobial (e.g., 47.6% for amoxicillin, 84% for ampicillin, 53.1% for cloxacillin, 61% for penicillin G, and 100% for cefoperazone and cefuroxime) [114].

Waste milk could also be dumped in the manure pit or lagoon effluent rather than being fed to calves [115]. Nevertheless, this practice results in environmental contamination with bacteria carrying AMR genes [112]. The screen-separated liquid fraction of dairy flushing wastewater that is pumped into a storage lagoon, known as dairy pond effluent, is frequently used as soil fertilizer for crop production [112]. In this regard, the presence of AMR genes and antimicrobial-resistant bacteria from waste milk will inevitably reach food products [116]. Effective management of waste milk remains as a hot topic for which few solutions are currently available.

**Table 1:** Research topic, species of interest, AMR genes, and relevant findings observed in key studies used in this review.

Topic	References	Species	AMR Genes	Findings
Global trends and epidemiology	[14]	*E. coli, K. pneumoniae*	-	Rapid increase in incidence rates of AMR among clinically important pathogens.
Prevalence and impact of *E. coli* infections	[19]	*E. coli*	ESBLs	Increasing detection rates of ESBLs among *E. coli* causing infections in humans.
*K. pneumoniae* epidemiology	[31,34,35]	*K. pneumoniae*	Carbapenemases, ESBLs	Widespread resistance to aminoglycosides, carbapenems, quinolones, and polymyxins.
Phylogenetic background and strain adaptation	[11,15]	*E. coli*	-	Clonal adaptation and increasing prevalence, especially of *E. coli* ST131, in humans.
Transmission of AMR via direct/indirect contact	[37,50,51,52,53]	*E. coli, K. pneumoniae*	ESBL, KPC-2	Increased risk of transmission among dairy farm workers; indirect transmission through food and environment; low evidence of direct transmission.
Pasteurization and raw milk consumption	[32,52,55,56]	*E. coli, Enterobacterales*	ESBL	Significant variation in raw milk consumption worldwide; association between raw milk and bacterial spread from cows to humans.
MGEs and AMR spread	[24,58,59]	*E. coli*	-	Emphasis on the role of MGEs in the spread of AMR; inconsistent strain distribution in bovine milk vs. human clinical strains.
Specific MGEs in mastitis	[39,60,61,62,63,64,65,66,67,68]	*E. coli, Enterobacterales*	*bla*_CTX-M_, *bla*_CMY-2_, *bla*_OXA-181_	ISEcp1 linked to *bla*_CTX-M_ genes and their dissemination; chromosomal and plasmid locations noted.
Plasmids and AMR genes	[39,76,77,78,79,80,81,82]	*E. coli, K. pneumoniae*	*bla*_CTX-M_, *bla*_TEM_, *bla*_SHV_, *bla*_CMY_	High mobility of IncI1, IncN, and IncF plasmids among isolates; widespread dissemination of ESBL genes like *bla*_CTX-M-15._
Integrons and gene cassettes	[60,71,72,73,74,75]	*E. coli, K. pneumoniae*	Various	High prevalence of class 1 integrons among isolates; need for full genetic context in epidemiological studies.
Surveillance and monitoring	[85,88,89]	*E. coli, Klebsiella* spp., other *Enterobacteriaceae*	ESBL/AmpC	Increasing resistance in *E. coli* isolates from cattle; presence of ESBL/AmpC producers.
Antimicrobial stewardship in mastitis	[90,91,92,95,98,99,100,105]	*E. coli, Klebsiella* spp.	-	Antimicrobial therapy for mastitis control is not always necessary: spontaneous cure or presence of non-susceptible pathogens. Varied pathogen responses influence treatment choices. Most mild and moderate CM caused by *E. coli* does not require antimicrobial treatment.
Environmental stewardship	[108,112,115,116]	*E. coli*, other *Enterobacteriaceae*	-	Presence of antimicrobial residue and multidrug-resistant E. coli in waste milk fed to calves and flushing wastewater dairy pond effluent as a potential disseminator AMR bacterium when used as soil fertilizer.

## 5. Future Directions

Certain knowledge gaps persist, highlighting possible further research directions. These include the following:

- The presence of KPC-2-positive, *K. pneumoniae* ST258 in the milk of cows affected by mastitis should be further investigated. It remains unclear what the possible human health implications associated with these strains are. Assessments of the potential risk of transmission from CM cases to humans, as well as possible transmission pathways, should be the focus.
- The ability of mastitis-causing MPEC and antimicrobial-resistant strains of the same genetic background as human isolates to effectively colonize and persist in human intestinal cells needs to be investigated. In this regard, the habit of consuming raw milk should be investigated as a potential risk factor for intestinal colonization by selected mastitis pathogens.
- The mechanisms of acquired AMR, including MGEs, among human and CM isolates within the same geographical regions should be contrasted to investigate the potential risk to public health associated with these elements. These will inform source attribution models and enable researchers to quantify human risks associated with antimicrobial-resistant coliforms in milk samples. Ideally, such investigations should also consider antimicrobials used in human medicine and in clinical mastitis treatment.
- The management of waste milk is still problematic, and technologies to effectively manage this product on farms should be developed.
- Finally, robust, integrated, and genomic-based systems should be designed for AMR surveillance in bovine mastitis and clinical human isolates. Ideally, these systems will also capture AMU data in animals and humans.
